# Animal Models of Tourette Syndrome—From Proliferation to Standardization

**DOI:** 10.3389/fnins.2016.00132

**Published:** 2016-03-31

**Authors:** Dorin Yael, Michal Israelashvili, Izhar Bar-Gad

**Affiliations:** The Leslie and Susan Goldschmied (Gonda) Multidisciplinary Brain Research Center, Bar-Ilan UniversityRamat-Gan, Israel

**Keywords:** Tourette syndrome, animal model, standardization, validation, striatum

## Abstract

Tourette syndrome (TS) is a childhood onset disorder characterized by motor and vocal tics and associated with multiple comorbid symptoms. Over the last decade, the accumulation of findings from TS patients and the emergence of new technologies have led to the development of novel animal models with high construct validity. In addition, animal models which were previously associated with other disorders were recently attributed to TS. The proliferation of TS animal models has accelerated TS research and provided a better understanding of the mechanism underlying the disorder. This newfound success generates novel challenges, since the conclusions that can be drawn from TS animal model studies are constrained by the considerable variation across models. Typically, each animal model examines a specific subset of deficits and centers on one field of research (physiology/genetics/pharmacology/etc.). Moreover, different studies do not use a standard lexicon to characterize different properties of the model. These factors hinder the evaluation of individual model validity as well as the comparison across models, leading to a formation of a fuzzy, segregated landscape of TS pathophysiology. Here, we call for a standardization process in the study of TS animal models as the next logical step. We believe that a generation of standard examination criteria will improve the utility of these models and enable their consolidation into a general framework. This should lead to a better understanding of these models and their relationship to TS, thereby improving the research of the mechanism underlying this disorder and aiding the development of new treatments.

## Tourette syndrome and the basal ganglia

Tourette syndrome (TS) is a neurodevelopmental disorder characterized by vocal and motor tics in the form of rapid, repetitive, non-rhythmic vocalizations or movements (American Psychiatric Association, 2013). The standard pharmacological treatment consists of the administration of antipsychotic drugs which act mainly as D2 dopamine receptor antagonists. However, this treatment has significant side effects and is typically not sufficient for complete tic suppression (Eddy et al., 2011). Unlike other motor disorders, tics are not completely involuntary. More than 90% of all TS patients report experiencing premonitory urges preceding the tic. These patients describe tics as voluntary actions which alleviate these uncomfortable urges (Leckman et al., 1993). While tics are the defining symptom of TS, most patients (>90%) suffer from additional symptoms classically associated with other disorders, such as attention deficit hyperactivity disorder (ADHD) and obsessive-compulsive behavior and disorder (OCD), each affecting roughly half of the patients (Freeman et al., 2000). Genetic factors were found to play a role in TS etiology (Price et al., 1985; Bertelsen et al., 2016). Nevertheless, most of the identified genes are rare, and to date no gene is known to have a major effect on TS etiology (Godar et al., 2014). The underlying pathophysiology of TS is currently unknown. Many different systems, brain regions and neuronal circuits are considered likely candidates, with most current studies linking the disorder to abnormalities in the cortico-basal ganglia (CBG) pathway.

The basal ganglia are a group of interconnected nuclei forming partially closed loops leading from most cortical areas back to frontal cortical areas. The loops are functionally divided into domains based on the cortical regions which send their input to the BG. The domains include the motor, associative (executive), and limbic areas. The role of the CBG pathway in TS has been hypothesized to be related with abnormal inhibition of undesired actions (Albin and Mink, 2006). This lack of inhibition has been attributed to local deficits within the striatum, which serves as the primary input nucleus of the BG. Most neurons in the striatum are the medium spiny projection neurons (MSNs) whose activity is modulated by interneurons such as GABAergic fast spiking interneurons (FSIs) and cholinergic tonically active neurons (TANs) (Kita et al., 1990; Bennett and Bolam, 1994; English et al., 2011) as well as by neuromodulatory afferents including dopaminergic, histaminergic, and adrenergic inputs (Holmberg et al., 1999; Surmeier et al., 2007; Ellender et al., 2011).

Converging evidence point to the involvement of the CBG loop, and specifically the striatum, in the pathology of TS: A small decrease in overall volume (Peterson et al., 2003) and a substantial reduction in the cell count of FSIs and TANs (Kalanithi et al., 2005; Kataoka et al., 2010) have been observed in the striatum of TS patients. Tic severity in early adulthood was found to be correlated with the extent of volume reduction of the caudate nucleus in childhood (Bloch et al., 2005). Further, correlations have been found between the severity of tics and the structural connectivity between the motor cortex and the striatum (Worbe et al., 2015), and between the supplementary motor area and the BG (Cheng et al., 2014). Abnormalities in neuronal transmission including decreased GABA_A_ receptor binding in the striatum (Lerner et al., 2012) and increased putamen dopamine release (Singer et al., 2002) have been reported in TS patients.

## Animal models of tourette syndrome

TS is a multifaceted disorder associated with a wide spectrum of clinical symptoms involving multiple underlying neuronal systems (Yael et al., 2015). In this perspective we focus on TS animal models related to the striatum since most findings from TS patients and most modern animal models are associated directly or indirectly with deficits within this brain region.

Motor and vocal tics, the primary symptom of TS, may be evoked by a disruption of GABAergic transmission within the striatum. Local microinjections of different GABA_A_ antagonists (such as bicuculline and picrotoxin) into the motor domain of the striatum have been shown to induce tics in both rodents (Marsden et al., 1975; Tarsy et al., 1978; Bronfeld et al., 2013b) and primates (Crossman et al., 1988; McCairn et al., 2009). The location of the disinhibition within the striatum determines the properties of the tics; injections in the motor striatum induce motor tics expressed in the body region associated with the somatotopic location of the striatal injection (Bronfeld et al., 2013b), whereas injections in the limbic striatum induce vocal tics (McCairn et al., 2016). Disinhibition in non-motor functional domains of the striatum induces behaviors similar to hyperactivity and compulsive symptoms (Worbe et al., 2009), thus exposing an intriguing link between tics and their comorbid symptoms. Additional support to the role of the striatum in TS and its comorbid disorders arise from a transgenic mouse model affecting the limbic cortico-striatal connectivity. This model demonstrates multiple symptoms such as OCD-like behaviors and sensorimotor gating deficits (Campbell et al., 1999; Nordstrom and Burton, 2002; Godar et al., 2015).

The identification of specific striatal neuronal subpopulations whose number is altered in TS (Kalanithi et al., 2005; Kataoka et al., 2010) inspired the development of animal models that target these subpopulations exclusively. Models have mimicked the selective suppression of the population of FSIs (using IEM-1460) thereby inducing abnormal movements (Gittis et al., 2011). The decline in the population of TANs has been modeled using viral-targeted cell ablation that leads to a highly specific reduction in this neuronal subpopulation in the dorsolateral striatum in mice (Xu et al., 2015). The ablation led to an increase in the expression of stereotypic behavior following stress and amphetamine treatment. However, although the animals displayed motor and behavioral abnormalities, no tics were observed in either model.

Other TS animal models are based on the dopaminergic model. This widely used model was originally related to other disorders such as schizophrenia, ADHD and OCD. Based on the “dopamine hypothesis,” which argues that the pathophysiology leading to TS involves hyper activation of the dopaminergic system (Singer et al., 1982), this model was associated with TS. Systemic (Randrup and Munkvad, 1967; Taylor et al., 2010) and intrastriatal (Kelley et al., 1988) administration of dopamine agonists (such as amphetamine and apomorphine) was shown to induce behavioral stereotypies and sensorimotor gating disruption (Mansbach et al., 1988; Swerdlow et al., 2003) but not motor or vocal tics. Dopamine induced behavioral stereotypies may be enhanced when other neuromodulator systems are disrupted, as has been recently illustrated in the histidine decarboxylase (HDC) knockout TS mouse model (Castellan Baldan et al., 2014).

## Validation of tourette syndrome animal models

The validation of animal models for human disorders is based upon three factors: face, predictive and construct validity. (1) Face validity is defined as the phenomenological similarity between the human clinical condition symptoms and symptoms expressed in the animal model. (2) Predictive validity refers to the ability of the model to predict some aspects of the disorder. Specifically, this validation is usually based on the extent to which the animals' response to medication can predict the human response. (3) Construct validity refers to the theoretical rationale of the model, based on the known pathophysiology of the disorder (Jinnah and Hess, 2005; Bronfeld et al., 2013a).

In TS animal models, assessment of face validity is complicated by the wide spectrum of features associated with the disorder due in part to the fact that TS lies in a gray area between movement disorders (based on the existence of motor tics) and psychiatric disorders (based on the premonitory urges and comorbid symptoms). The primary feature associated with the movement disorder aspect of TS is the ability to induce tic-like movements. Currently, the striatal disinhibition model is the only one expressing motor tic-like movements (Marsden et al., 1975; Crossman et al., 1988; McCairn et al., 2009; Bronfeld et al., 2013b) and/or vocal tic-like sounds (McCairn et al., 2016). Other animal models typically elicit other forms of abnormal movements such as dyskinesia and dystonia (Gittis et al., 2011). Assessing TS as a psychiatric disorder complicates the evaluation of face validity. It is impossible to directly assess the existence of premonitory urges in animals; however, it was suggested these can reflect deficits in sensory motor gating (Swerdlow et al., 1999). Thus, indirectly these urges can be assessed by the pre-pulse inhibition (PPI) paradigm. Using PPI, deficits in the sensory motor gating have been reported in several dopamine related animal models (Mansbach et al., 1988; Castellan Baldan et al., 2014) but have not been tested in other models. Another aspect of TS is the high rates of comorbid conditions. Dopaminergic, cholinergic (TANs) and HDC models (subsequent to stress and/or amphetamine injection) were found to show an increase in stereotypic behaviors (Randrup et al., 1963; Kelley et al., 1988; Castellan Baldan et al., 2014; Xu et al., 2015) whereas the striatal disinhibition model demonstrated both hyperactivity and stereotypy following manipulation of non-motor (limbic and associative) areas in the striatum (Worbe et al., 2009).

The predictive validation of TS animal models is restricted by the non-specific medication for TS which is mostly based on responses to antipsychotic drugs (Shapiro and Shapiro, 1968). Due to the common definition, the dopaminergic animal models have a high predictive validity, as this model is based on the effectiveness of these drugs. Other models have not been explicitly tested for response to antipsychotics as well as to other drug treatments.

Evaluating the construct validity of TS animal models is currently speculative because the underlying pathophysiology of TS is still unclear. Typically the construct validity of TS animal models is based on their relationship to the small subset of currently known differences identified in TS patients compared to controls. Striatal animal models have been linked to current evidence from human studies, including dopamine dysfunction (Singer et al., 1991, 2002; Cheon et al., 2004; Minzer et al., 2004; Steeves et al., 2010), genetic abnormalities in a small subpopulation of TS patients (HDC model; Ercan-Sencicek et al., 2010), and a reduction in the cell count of striatal FSIs and TANs (Kalanithi et al., 2005; Kataoka et al., 2010). Another approach to assessing the construct validity of TS animal models is their relationship to theoretical functional models of information processing in the BG in both normal and pathological states. The “box and arrow” model of the BG describes their function based on their main anatomical connectivity (Albin et al., 1989; DeLong, 1990). According to this model, dopaminergic innervation to the striatum modulates striatal activity and consequently increases the overall cortical activation, leading to hyperkinetic symptoms. Consistent with this model, the dopaminergic model yields increased movement in the form of stereotypic behavior (Randrup and Munkvad, 1967; Kelley et al., 1988). This behavior was also observed in HDC knockout (Castellan Baldan et al., 2014) and TAN ablated (Xu et al., 2015) mouse models of TS which demonstrated enhanced stereotypic behavior in response to dopamine agonists. The “action selection” model contemplates that the BG chooses a single action while inhibiting competing actions (Mink, 1996). A loss of inhibition in a specific area within the striatum would thus prevent the selection process (Mink, 2001). This coincides with the animal model of focal disinhibition of the striatum using local blocking of GABA which prevents input from all inhibitory sources, including FSIs and neighboring MSNs (McCairn et al., 2009; Worbe et al., 2013). This functional model may also explain the behavioral and neuronal effects observed in the animal model based on selective suppression of FSIs (Gittis et al., 2011).

## Standardization of tourette syndrome animal models

Over the last decade, rapid progress has been made in TS animal models studies, leading to a proliferation of novel models. Accumulating results from imaging, genetic, and anatomic studies performed on TS patients, provided solid foundation for the development of novel models with high construct validity, such as the TANs, FSIs, and HDC models. In addition, animal models which were previously attributed to other disorders have been recently considered valid models of TS. These include the striatal disinhibition model previously attributed to myoclonus (Marsden et al., 1975) and the dopaminergic models previously attributed to OCD and/or schizophrenia (Swerdlow and Geyer, 1998; Korff and Harvey, 2006). The use of different species of animals has made it possible to investigate multiple properties of the disorder by utilizing the relative advantages of each species. Mouse studies have enabled the investigation of genetic manipulation, rat studies explore the relationship between pharmacology, physiology, and behavior, and primate studies serve the study of complex behaviors linking the motor and psychiatric aspects of the disorder. Studies utilizing animal models significantly improved our understanding of the underlying mechanisms of specific properties of TS. For example, key questions such as “when” and “where” tics are expressed were recently addressed in animal model studies (Bronfeld et al., 2013b; Israelashvili and Bar-Gad, 2015). Similarly, data pointing to a common pathophysiology of TS and its comorbid conditions were experimentally supported when the same manipulation yielded a variety of behavioral symptoms (Worbe et al., 2009). This progress has led to a situation where finally basic and clinical science can co-contribute to the study of this disorder.

This major progress generates new challenges faced by TS animal models studies. The complexity of TS research which results from the myriad of symptoms and the fact that the pathophysiology leading to the disorder is still largely unknown have resulted in high variability in the study of mechanisms and behavioral symptoms in animal models of TS. Naturally, each of the current animal models focuses on a small subset of symptoms associated with the disorder which are examined using specific, non-standard and non-overlapping tests. Typically, these early-stage models were developed and studied by teams with specific expertise such as pharmacology, electrophysiology and genetics, thus creating a situation in which different models are studied with a high degree of focus into one field with significantly less effort in others. As a result, under the broad title of a “TS animal model study,” a variety of models exist, that vary widely as regards to the scientific basis, methods used and the features examined. This issue is evident even when different models examine the same feature. The description of motor deficits does not adhere to an accepted classification, which leads to unclear (and in some cases ill-defined) definitions such as tic-like movement, tic-like stereotypy, and tic-like dyskinesia, without a robust kinematic evaluation. While this variability may contribute to a better understanding and characterization of different aspects of the disorder, it hinders the comparison across results of different models and the evaluation of the relevance, uniqueness, and contribution of each model. Furthermore, the use of this wide nomenclature is not necessarily supported by a large variety of underlying behavioral symptoms. A standardization of the examination criteria of TS animal models could help overcome these challenges.

Standardization is a widely used procedure in multiple fields to ensure consistency and comparability between processes or products. Evaluation criteria defined in standard protocols assist in the assessment of relevant information and allow uniformity within and between different users. In the clinic the necessity of standard protocols for diagnosis and treatment was raised for TS and other disorders, leading to the development of multiple standardized guidelines. The consistency and comparability enabled by these guidelines are highly beneficial in terms of the ability to properly diagnose and treat individual patients. More broadly it provides a uniform database enabling future study and the existence of a worldwide discourse. The reasons that led to the establishment of guidelines in the clinic apply to the development of standardized assessment of TS animal models that are still lacking in the field.

A standardization process defining the gold standard for the evaluation of TS animal model will improve the utility of these models and their use in both basic science as well as drug and treatment discovery. Standardized parameters will explicitly define the major components of a TS animal model evaluation, by providing an organized list detailing the range of characteristics of a valid TS animal model. In addition, a standardization process will allow an evaluation of the studied model's coverage, pointing to features that were studied using a specific model and those that were not. The standard definitions arrived at through this process will provide a clear differentiation between behavioral, pharmacological or physiological characteristics which are qualitatively different. These definitions will provide well-defined guidelines for classification of parameters while avoiding a use of fuzzy definitions. Thus, it will enable categorizing behaviors into either different categories if these behaviors resemble distinct symptoms or into a merged category if they resemble similar symptoms. This common terminology will allow both the evaluation of each model by standardized categories, and more importantly, a comparison between different models using the same vocabulary. The process of standardization in TS animal model research will help combine information from different studies into a general framework describing the mechanisms and their behavioral outcomes. It will enable a transition in TS research from small, local attempts typically confined to a single lab into a global effort. The development and implementation of such standards by a single lab or a small group of cooperating labs is prone to generate a partial picture biased by the inherent properties of this group. Thus, the standard criteria for TS animal model evaluation should be developed by a diverse committee including experts from various fields, reflecting a broad and comprehensive perspective, including both basic scientists developing and studying animal models, scientists conducting studies with TS patients and the clinicians working with these patients. This committee should be responsible for both writing the guidelines and maintaining a worldwide database summarizing results from different studies. We believe that such an effort should be managed by research-supporting international associations such as the Tourette Association of America (TAA) and/or the European Society for the Study of Tourette Syndrome (ESSTS), which hold the ability to direct international efforts. This standardization process, previously shown to be highly beneficial in other fields, has the potential to translate the rapidly accumulating results into a comprehensive framework for experimental studies of TS.

## Author contributions

All authors listed, have made substantial, direct and intellectual contribution to the work, and approved it for publication.

## Funding

The preparation of this manuscript was supported by an Israel Science Foundation (ISF) grant (743/13) and a Tourette Syndrome Association (TSA) grant.

### Conflict of interest statement

The authors declare that the research was conducted in the absence of any commercial or financial relationships that could be construed as a potential conflict of interest. The reviewer BH and handling Editor declared an ongoing co-authorship, and the handling Editor states that the process nevertheless met the standards of a fair and objective review.

## References

[B1] Albin R. L. Mink J. W. (2006). Recent advances in Tourette syndrome research. Trends Neurosci. 29, 175–182. 10.1016/j.tins.2006.01.001 16430974

[B2] Albin R. L. Young A. B. Penney J. B. (1989). The functional anatomy of basal ganglia disorders. Trends Neurosci. 12, 366–375. 10.1016/0166-2236(89)90074-X 2479133

[B3] American Psychiatric Association (2013). DSM-5. Arlington, TX: American Psychiatric Association Publishing.

[B4] Bennett B. D. Bolam J. P. (1994). Synaptic input and output of parvalbumin-immunoreactive neurons in the neostriatum of the rat. Neuroscience 62, 707–719. 10.1016/0306-4522(94)90471-5 7870301

[B5] Bertelsen B. Stefánsson H. Riff Jensen L. Melchior L. Mol Debes N. Groth C.. (2016). Association of AADAC deletion and gilles de la tourette syndrome in a large European cohort. Biol. Psychiatry 79, 383–391. 10.1016/j.biopsych.2015.08.027 26444075

[B6] Bloch M. H. Leckman J. F. Zhu H. Peterson B. S. (2005). Caudate volumes in childhood predict symptom severity in adults with Tourette syndrome. Neurology 65, 1253–1258. 10.1212/01.wnl.0000180957.98702.69 16247053 PMC2367161

[B7] Bronfeld M. Israelashvili M. Bar-Gad I. (2013a). Pharmacological animal models of Tourette syndrome. Neurosci. Biobehav. Rev. 37, 1101–1119. 10.1016/j.neubiorev.2012.09.010 23089155

[B8] Bronfeld M. Yael D. Belelovsky K. Bar-Gad I. (2013b). Motor tics evoked by striatal disinhibition in the rat. Front. Syst. Neurosci. 7:50. 10.3389/fnsys.2013.00050 24065893 PMC3776161

[B9] Campbell K. M. de Lecea L. Severynse D. M. Caron M. G. McGrath M. J. Sparber S. B.. (1999). OCD-Like behaviors caused by a neuropotentiating transgene targeted to cortical and limbic D1+ neurons. J. Neurosci. 19, 5044–5053. 10366637 10.1523/JNEUROSCI.19-12-05044.1999 PMC6782675

[B10] Castellan Baldan L. Williams K. A. Gallezot J. D. Pogorelov V. Rapanelli M. Crowley M.. (2014). Histidine decarboxylase deficiency causes tourette syndrome: parallel findings in humans and mice. Neuron 81, 77–90. 10.1016/j.neuron.2013.10.052 24411733 PMC3894588

[B11] Cheng B. Braass H. Ganos C. Treszl A. Biermann-Ruben K. Hummel F. C.. (2014). Altered intrahemispheric structural connectivity in Gilles de la Tourette syndrome. NeuroImage Clin. 4, 174–181. 10.1016/j.nicl.2013.11.011 24371800 PMC3872720

[B12] Cheon K.-A. Ryu Y.-H. Namkoong K. Kim C.-H. Kim J.-J. Lee J. D. (2004). Dopamine transporter density of the basal ganglia assessed with [123I]IPT SPECT in drug-naive children with Tourette's disorder. Psychiatry Res. 130, 85–95. 10.1016/j.pscychresns.2003.06.001 14972371

[B13] Crossman A. R. Mitchell I. J. Sambrook M. A. Jackson A. (1988). Chorea and myoclonus in the monkey induced by gamma-aminobutyric acid antagonism in the lentiform complex. Brain 111, 1211–1233. 10.1093/brain/111.5.1211 3179691

[B14] DeLong M. R. (1990). Primate models of movement disorders of basal ganglia origin. Trends Neurosci. 13, 281–285. 10.1016/0166-2236(90)90110-V 1695404

[B15] Eddy C. M. Rickards H. E. Cavanna A. E. (2011). Treatment strategies for tics in Tourette syndrome. Ther. Adv. Neurol. Disord. 4, 25–45. 10.1177/1756285610390261 21339906 PMC3036957

[B16] Ellender T. J. Huerta-Ocampo I. Deisseroth K. Capogna M. Bolam J. P. (2011). Differential modulation of excitatory and inhibitory striatal synaptic transmission by histamine. J. Neurosci. 31, 15340–15351. 10.1523/JNEUROSCI.3144-11.2011 22031880 PMC6703542

[B17] English D. F. Ibanez-Sandoval O. Stark E. Tecuapetla F. Buzsáki G. Deisseroth K.. (2011). GABAergic circuits mediate the reinforcement-related signals of striatal cholinergic interneurons. Nat. Neurosci. 15, 123–130. 10.1038/nn.2984 22158514 PMC3245803

[B18] Ercan-Sencicek A. G. Stillman A. A. Ghosh A. K. Bilguvar K. O'Roak B. J. Mason C. E.. (2010). L-histidine decarboxylase and Tourette's syndrome. N. Engl. J. Med. 362, 1901–1908. 10.1056/NEJMoa0907006 20445167 PMC2894694

[B19] Freeman R. D. Fast D. K. Burd L. Kerbeshian J. Robertson M. M. Sandor P. (2000). An international perspective on Tourette syndrome: selected findings from 3,500 individuals in 22 countries. Dev. Med. Child Neurol. 42, 436–447. 10.1017/S0012162200000839 10972415

[B20] Gittis A. H. Leventhal D. K. Fensterheim B. A. Pettibone J. R. Berke J. D. Kreitzer A. C. (2011). Selective inhibition of striatal fast-spiking interneurons causes dyskinesias. J. Neurosci. 31, 15727–15731. 10.1523/JNEUROSCI.3875-11.2011 22049415 PMC3226784

[B21] Godar S. C. Mosher L. J. Di Giovanni G. Bortolato M. (2014). Animal models of tic disorders: a translational perspective. J. Neurosci. Methods 238, 54–69. 10.1016/j.jneumeth.2014.09.008 25244952 PMC4345166

[B22] Godar S. C. Mosher L. J. Strathman H. J. Gochi A. M. Jones C. M. Fowler S. C.. (2015). *The D1CT-7* mouse model of Tourette syndrome displays sensorimotor gating deficits in response to spatial confinement. Br. J. Pharmacol. [Epub ahead of print]. 10.1111/bph.13243 26171666 PMC4908200

[B23] Holmberg M. Scheinin M. Kurose H. Miettinen R. (1999). Adrenergic alpha2C-receptors reside in rat striatal GABAergic projection neurons: comparison of radioligand binding and immunohistochemistry. Neuroscience 93, 1323–1333. 10.1016/S0306-4522(99)00260-2 10501456

[B24] Israelashvili M. Bar-Gad I. (2015). Corticostriatal divergent function in determining the temporal and spatial properties of motor tics. J. Neurosci. 35, 16340–16351. 10.1523/JNEUROSCI.2770-15.2015 26674861 PMC4679818

[B25] Jinnah H. A. Hess E. J. (2005). Assessment of movement disorders in rodents, in Animal Models of Movement Disorders, ed LeDoux M. (Amsterdam: Elsevier Academic Press), 55–72.

[B26] Kalanithi P. S. A. Zheng W. Kataoka Y. DiFiglia M. Grantz H. Saper C. B.. (2005). Altered parvalbumin-positive neuron distribution in basal ganglia of individuals with Tourette syndrome. Proc. Natl. Acad. Sci. U.S.A. 102, 13307–13312. 10.1073/pnas.0502624102 16131542 PMC1201574

[B27] Kataoka Y. Kalanithi P. S. A. Grantz H. Schwartz M. L. Saper C. Leckman J. F.. (2010). Decreased number of parvalbumin and cholinergic interneurons in the striatum of individuals with Tourette syndrome. J. Comp. Neurol. 518, 277–291. 10.1002/cne.22206 19941350 PMC2846837

[B28] Kelley A. E. Lang C. G. Gauthier A. M. (1988). Induction of oral stereotypy following amphetamine microinjection into a discrete subregion of the striatum. Psychopharmacology (Berl). 95, 556–559. 10.1007/BF00172976 3145527

[B29] Kita H. Kosaka T. Heizmann C. W. (1990). Parvalbumin-immunoreactive neurons in the rat neostriatum: a light and electron microscopic study. Brain Res. 536, 1–15. 10.1016/0006-8993(90)90002-S 2085740

[B30] Korff S. Harvey B. H. (2006). Animal models of obsessive-compulsive disorder: rationale to understanding psychobiology and pharmacology. Psychiatr. Clin. North Am. 29, 371–390. 10.1016/j.psc.2006.02.007 16650714

[B31] Leckman J. F. Walker D. E. Cohen D. J. (1993). Premonitory urges in Tourette's syndrome. Am. J. Psychiatry 150, 98–102. 10.1176/ajp.150.1.98 8417589

[B32] Lerner A. Bagic A. Simmons J. M. Mari Z. Bonne O. Xu B.. (2012). Widespread abnormality of the γ-aminobutyric acid-ergic system in Tourette syndrome. Brain 135, 926–1936. 10.1093/brain/aws104 22577221 PMC3359755

[B33] Mansbach R. S. Geyer M. A. Braff D. L. (1988). Dopaminergic stimulation disrupts sensorimotor gating in the rat. Psychopharmacology (Berl). 94, 507–514. 10.1007/BF00212846 3131796

[B34] Marsden C. D. Meldrum B. S. Pycock C. Tarsy D. (1975). Focal myoclonus produced by injection of picrotoxin into the caudate nucleus of the rat. J. Physiol. (Lond). 246, 96P. 1142296

[B35] McCairn K. W. Bronfeld M. Belelovsky K. Bar-Gad I. (2009). The neurophysiological correlates of motor tics following focal striatal disinhibition. Brain 132, 2125–2138. 10.1093/brain/awp142 19506070

[B36] McCairn K. W. Nagai Y. Hori Y. Ninomiya T. Kikuchi E. Lee J.-Y.. (2016). A primary role for nucleus accumbens and related limbic network in vocal tics. Neuron 89, 300–307. 10.1016/j.neuron.2015.12.025 26796690

[B37] Mink J. W. (1996). The basal ganglia: focused selection and inhibition of competing motor programs. Prog. Neurobiol. 50, 381–425. 10.1016/S0301-0082(96)00042-1 9004351

[B38] Mink J. W. (2001). Basal ganglia dysfunction in Tourette's syndrome: a new hypothesis. Pediatr. Neurol. 25, 190–198. 10.1016/S0887-8994(01)00262-4 11587872

[B39] Minzer K. Lee O. Hong J. J. Singer H. S. (2004). Increased prefrontal D2 protein in Tourette syndrome: a postmortem analysis of frontal cortex and striatum. J. Neurol. Sci. 219, 55–61. 10.1016/j.jns.2003.12.006 15050438

[B40] Nordstrom E. J. Burton F. H. (2002). A transgenic model of comorbid Tourette's syndrome and obsessive-compulsive disorder circuitry. Mol. Psychiatry 7, 617–625. 10.1038/sj.mp.4001144 12140785

[B41] Peterson B. S. Thomas P. Kane M. J. Scahill L. Zhang H. Bronen R.. (2003). Basal Ganglia volumes in patients with Gilles de la Tourette syndrome. Arch. Gen. Psychiatry 60, 415–424. 10.1001/archpsyc.60.4.415 12695320

[B42] Price R. A. Kidd K. K. Cohen D. J. Pauls D. L. Leckman J. F. (1985). A twin study of Tourette syndrome. Arch. Gen. Psychiatry 42, 815–820. 10.1001/archpsyc.1985.01790310077011 3860194

[B43] Randrup A. Munkvad I. (1967). Stereotyped activities produced by amphetamine in several animal species and man. Psychopharmacologia 11, 300–310. 10.1007/BF00404607 4968376

[B44] Randrup A. Munkvad I. Udsen P. (1963). Adrenergic mechanisms and amphetamine induced abnormal behaviour. Acta Pharmacol. Toxicol. (Copenh). 20, 145–157. 10.1111/j.1600-0773.1963.tb01731.x 14060772

[B45] Shapiro A. K. Shapiro E. (1968). Treatment of Gilles de la Tourette's Syndrome with haloperidol. Br. J. Psychiatry 114, 345–350. 10.1192/bjp.114.508.345 4384341

[B46] Singer H. S. Butler I. J. Tune L. E. Seifert W. E. Jr. Coyle J. T. (1982). Dopaminergic dsyfunction in Tourette syndrome. Ann. Neurol. 12, 361–366. 10.1002/ana.410120408 6184010

[B47] Singer H. S. Hahn I. H. Moran T. H. (1991). Abnormal dopamine uptake sites in postmortem striatum from patients with Tourette's syndrome. Ann. Neurol. 30, 558–562. 10.1002/ana.410300408 1838678

[B48] Singer H. S. Szymanski S. Giuliano J. Yokoi F. Dogan A. S. Brasic J. R.. (2002). Elevated intrasynaptic dopamine release in Tourette's syndrome measured by PET. Am. J. Psychiatry 159, 1329–1336. 10.1176/appi.ajp.159.8.1329 12153825

[B49] Steeves T. D. L. Ko J. H. Kideckel D. M. Rusjan P. Houle S. Sandor P.. (2010). Extrastriatal dopaminergic dysfunction in tourette syndrome. Ann. Neurol. 67, 170–181. 10.1002/ana.21809 20225192

[B50] Surmeier D. J. Ding J. Day M. Wang Z. Shen W. (2007). D1 and D2 dopamine-receptor modulation of striatal glutamatergic signaling in striatal medium spiny neurons. Trends Neurosci. 30, 228–235. 10.1016/j.tins.2007.03.008 17408758

[B51] Swerdlow N. R. Braff D. L. Geyer M. A. (1999). Cross-species studies of sensorimotor gating of the startle reflex. Ann. N. Y. Acad. Sci. 877, 202–216. 10.1111/j.1749-6632.1999.tb09269.x 10415651

[B52] Swerdlow N. R. Geyer M. A. (1998). Using an animal model of deficient sensorimotor gating to study the pathophysiology and new treatments of schizophrenia. Schizophr. Bull. 24, 285–301. 10.1093/oxfordjournals.schbul.a033326 9613626

[B53] Swerdlow N. R. Shoemaker J. M. Platten A. Pitcher L. Goins J. Crain S. (2003). Heritable differences in the effects of amphetamine but not DOI on startle gating in albino and hooded outbred rat strains. Pharmacol. Biochem. Behav. 75, 191–197. 10.1016/S0091-3057(03)00078-9 12759127

[B54] Tarsy D. Pycock C. J. Meldrum B. S. Marsden C. D. (1978). Focal contralateral myoclonus produced by inhibition of GABA action in the caudate nucleus of rats. Brain 101, 143–162. 10.1093/brain/101.1.143 638722

[B55] Taylor J. L. Rajbhandari A. K. Berridge K. C. Aldridge J. W. (2010). Dopamine receptor modulation of repetitive grooming actions in the rat: potential relevance for Tourette syndrome. Brain Res. 1322, 92–101. 10.1016/j.brainres.2010.01.052 20114036 PMC2858339

[B56] Worbe Y. Baup N. Grabli D. Chaigneau M. Mounayar S. McCairn K.. (2009). Behavioral and movement disorders induced by local inhibitory dysfunction in primate striatum. Cereb. Cortex 19, 1844–1856. 10.1093/cercor/bhn214 19068490

[B57] Worbe Y. Marrakchi-Kacem L. Lecomte S. Valabregue R. Poupon F. Guevara P.. (2015). Altered structural connectivity of cortico-striato-pallido-thalamic networks in Gilles de la Tourette syndrome. Brain 138, 472–482. 10.1093/brain/awu311 25392196 PMC4306818

[B58] Worbe Y. Sgambato-Faure V. Epinat J. Chaigneau M. Tandé D. François C.. (2013). Towards a primate model of Gilles de la Tourette syndrome: anatomo-behavioural correlation of disorders induced by striatal dysfunction. Cortex 49, 1126–1140. 10.1016/j.cortex.2012.08.020 23040317

[B59] Xu M. Kobets A. Du J.-C. Lennington J. Li L. Banasr M.. (2015). Targeted ablation of cholinergic interneurons in the dorsolateral striatum produces behavioral manifestations of Tourette syndrome. Proc. Natl. Acad. Sci. U.S.A. 112, 893–898. 10.1073/pnas.1419533112 25561540 PMC4311862

[B60] Yael D. Vinner E. Bar-Gad I. (2015). Pathophysiology of tic disorders. Mov. Disord. 30, 1171–1178. 10.1002/mds.26304 26179434

